# Simulation-guided outlet valve repair: early integration of computationally-derived principles of free-edge length in bicuspidization repair

**DOI:** 10.3389/fcvm.2026.1957668

**Published:** 2026-09-29

**Authors:** Perry Seo Choi, Alexander D. Kaiser, Riya Nilkant, Amit Sharir, Umar Siddiqi, Hannah L. McMullen, Alison L. Marsden, Michael R. Ma

**Affiliations:** 1 Department of Cardiothoracic Surgery, Division of Pediatric Cardiac Surgery, Stanford University, Stanford, CA, United States; 2 Stanford Maternal and Child Health Research Institute, Stanford, CA, United States; 3 Cardiovascular Institute, Stanford University, Stanford, CA, United States; 4 Department of Pediatrics, Division of Pediatric Cardiology, Stanford University, Stanford, CA, United States; 5 Department of Bioengineering, Stanford University, Stanford, CA, United States; 6 Institute for Computational & Mathematical Engineering, Stanford University, Stanford, CA, United States

**Keywords:** aortic valve, aortic valve repair, bicuspidization, congential heart disease, truncal valve repair, computational modeling, bioengineering

## Abstract

**Background:**

Symmetric bicuspidization repair for outlet valve pathologies relies on the surgeon's subjective assessment. Recent computational and ex-vivo work suggests sizing free-edge length to 1.57 times aortic diameter is optimal. This principle was applied clinically at our institution, and this study compares post-implementation outcomes with historical results.

**Methods:**

All patients with attempted bicuspidization from 2011 to 2025 were queried. Primary outcome was freedom from valve-related reoperation or valve replacement. Secondary outcomes were cross-clamp time, number of bypass runs, mean transvalvular gradient, and regurgitation grade. Kaplan–Meier curves were generated for primary outcome with log-rank comparison. Secondary outcomes were compared with Wilcoxon rank-sum test.

**Results:**

At median age 5.9 years, fourteen patients underwent symmetric bicuspidization (9 historical, 5 simulation-guided). Valve configuration included unicuspid (*n* = 2), bicuspid (*n* = 6), tricuspid (*n* = 4), and quadricuspid (*n* = 2). Repair indication was predominantly regurgitation (*n* = 13/14, 93%). Five (56%) historical patients underwent reoperation compared with zero simulation-guided patients (median follow-up 948 vs. 455 days). Three (33%) historical patients required repeat bypass run, compared to zero simulation-guided. Cross-clamp time was not different between groups (72 vs. 89, *p* = 0.74). Acute and latest postoperative mean gradients were also not significantly different (acute: 13.5 vs. 9.6, *p* = 0.14; latest: 12.4 vs. 3.3, *p* = 0.08), though gradient variability was two to four times lower in the simulation-guided group.

**Conclusions:**

This exploratory study presents promising early outcomes after prespecified, standardized sizing of the free-edge length in symmetric bicuspidization repair. Simulation-guided design can be applied in complex valve repair and may enhance early outcomes.

## Introduction

Bicuspidization repair has proven to be an effective repair strategy when applied to severe congenital aortic or truncal valve pathologies (1–3). In a previous simulation-based study, we demonstrated that undersizing free-edge length creates significant transvalvular gradient in computational models, and that a free-edge length-to-aortic diameter (FELAD) ratio roughly 1.57 appears ideal (Figure 1, top) (4). Subsequently, these findings were validated in an ex-vivo porcine model, which confirmed stenosis with FELAD <1.2 and improved hemodynamics with FELAD 1.57, while also revealing adverse flow profiles with FELAD >1.8 (5). As such, our institution began implementing a simulation-guided, standardized approach to symmetric bicuspidization repair in 2023, setting the free-edge length of the bicuspidized valve to a prespecified FELAD ratio of 1.57. The present study examines postoperative outcomes for the post-implementation, simulation-guided group and compares to our pre-implementation, historical results.

**Figure 1:**
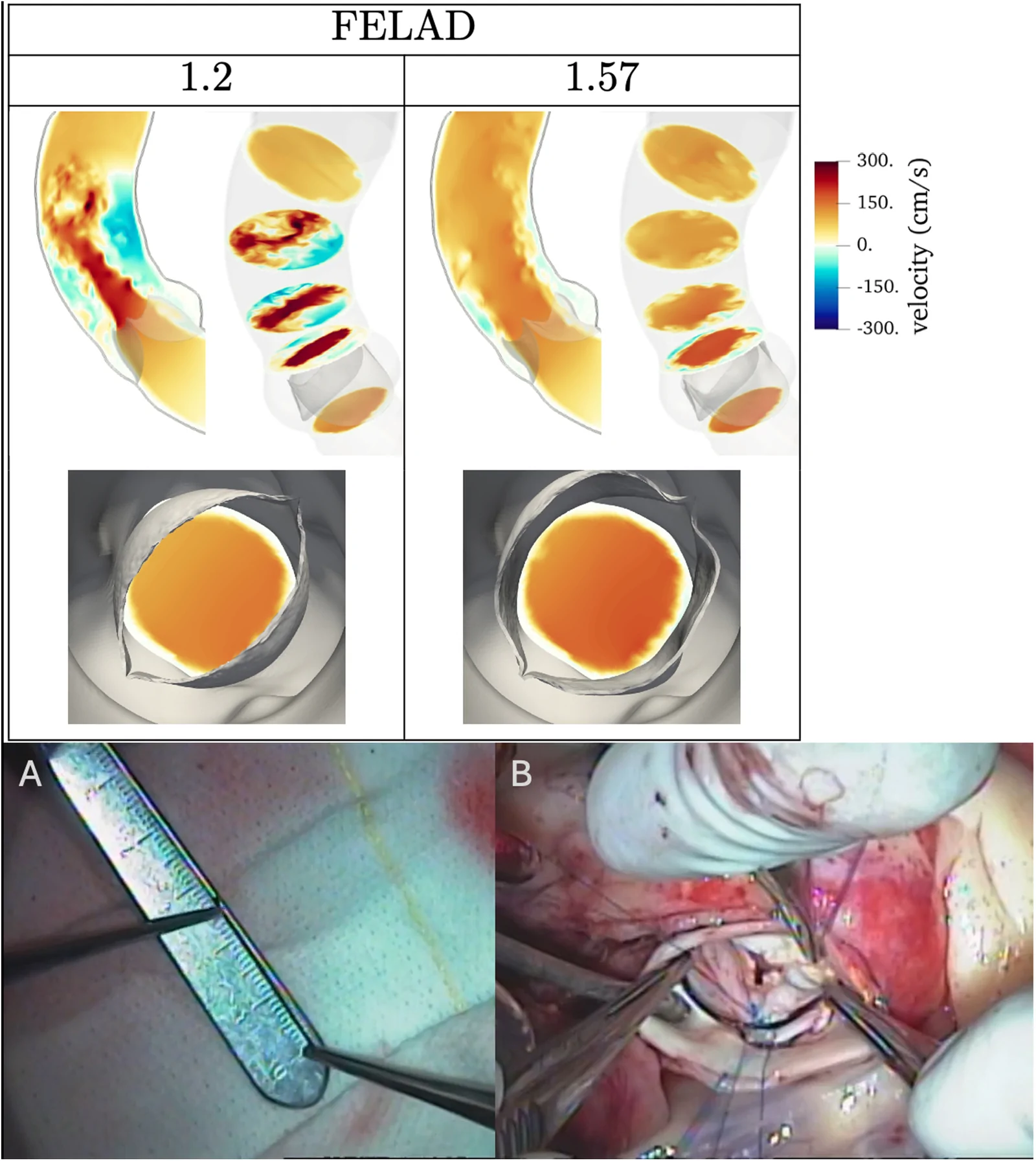
Translation from simulation to operating room. Top) Computational flow simulations demonstrating stenosis at peak transvalvular gradient in mid systole of bicuspidized outlet valves at free-edge length 1.2 times the aortic annulus (peak gradient 23 mmHg) and relief of stenosis at values of 1.57 (peak gradient 6 mmHg). Panels show the vertical component of velocity, axial velocity on slices and an *en face* view. **A)** Intraoperative image of surgeon confirming the length of the cut silk suture matches the target length, calculated as 1.57 times the clinically measured sinotubular junction diameter. **B)** The surgeon uses the pre-measured silk suture from panel A to confirm that the target free-edge length of the bicuspidized valve has been achieved. If discrepant, the surgeon will further modify the free-edge length of the repair.

Modeling and simulation have been previously applied to cardiac procedures. A pioneering computational study on the Fontan procedure predicted an offset postoperative geometry was more favorable; this concept has subsequently clinically adopted (6). Simulations predicted a novel Y-graft design would improve Fontan performance (7) and subsequently performed prospectively in patients (8). Simulations have also been applied prospectively for planning of Fontan repair with interrupted inferior vena cava (9). Commercial entities have successfully used simulations for coronary flow (10) and valve prosthetics (11). Geometric modeling studies have been used for planning of complex valve repair (12). A recent study used anatomical measurements to create guidelines with ratios similar to this study with promising initial results (13). There appear to be, however, few studies including fluid-structure interaction simulation of heart valves that have directly influenced valve repair.

## Methods

This is a single-institution retrospective analysis of patients who underwent attempted symmetric bicuspidization repair for systemic outlet valve pathology from 2011 to 2025. Primary outcome was freedom from valve-related reoperation or valve replacement. Kaplan–Meier curves were generated for primary outcome with log-rank comparison. Secondary outcomes were cross-clamp time, number of bypass runs, and both acute and latest postoperative mean transvalvular gradient and regurgitation grade. Due to variability of post-operative gradients in the hospital phase, the acute mean gradient was determined as the maximum among all documented echocardiographic values over the first two postoperative months. Acute regurgitation was similarly reported as the maximum grade reported during first two postoperative months in ordinal form (trace = 0.5, mild = 1, mild-moderate = 1.5, moderate = 2, moderate-severe = 2.5, severe = 3). This project was approved by the Institutional Review Board (protocol 52170, approved on 11/20/2024). To assess potential confounding by patch augmentation, a sensitivity analysis repeated the primary-outcome and postoperative-gradient comparisons after excluding the three historical patients who underwent autologous pericardial patch augmentation.

### Surgical implementation

For the simulation-guided group, the prespecified free-edge length was calculated by measuring the sinotubular junction diameter and then multiplying by 1.57. This length was confirmed by cutting a silk suture to this prespecified length and then ensuring the free-edge of the newly repaired valve matched the length of this silk suture (Figures 1A, B). The sinotubular junction (STJ) was used as the clinical denominator because prior simulations treated the virtual basal ring and STJ diameters as equal and used the STJ as the more clinically accessible reference. If the reconstructed free-edge length exceeded the target derived from the measured diameter, leaflet tissue was plicated to shorten free-edge length. If free-edge length was insufficient, patch augmentation was considered when appropriate, though patch material was avoided whenever possible.

### Simulation methods

The simulation workflow and results have been described in detail previously (4) and are briefly reviewed here. While the cohort in this manuscript focuses on pediatric repair, these methods are applicable for pediatric and adult patients alike. Fluid-structure interaction simulations, which model the coupled dynamics of the valve and blood, were performed via the immersed boundary method (14) in the open-source framework IBAMR (15) with fluid domain mesh width 0.5 mm. Valve models were constructed with a generative, elasticity-based design approach (16). Models were constructed with reference free-edge length 1.2 and 1.57 times the annular diameter, which here was equal to the sinotubular junction diameter. In this method, differential equations of mechanical equilibrium were solved to predict the loaded configuration of the valve with each desired geometry. The reference or resting configuration was then computed from the predicted loaded configuration, and stiffnesses were scaled to support the predicted load. Valve tissue was model with elastic fiber-based material properties, and this process produced comparable stiffnesses to native aortic valve tissue (17). Code for model generation is open-source and freely available (github.com/alexkaiser/heart_valves). An anonymized CT scan was obtained, and image segmentation was performed in SimVascular (18). The segmented domain included the left ventricular outflow tract to the ascending aorta, stopping slightly before the arch vessels. A mesh for the aorta-lumen boundary was generated with target edge size 0.25 mm. The aorta geometry was held approximately rigid with “target points,” stiff linear springs that maintain a nearly constant position. Physiological, time-dependent waveforms were applied via pressure boundary conditions. Ventricular pressure at the fluid domain inlet was determined by a time-varying elastance model (19) with target cardiac output of 5.6 L/min under conditions of negligible valve resistance and heart rate 75 beats per minute. Aortic pressure at the fluid domain outlet was controlled by resistor-capacitor-resistor boundary condition with values tuned independently for nominal pressure of 120/80.

## Results

At median age 5.9 (IQR 2.7,11.9) years, fourteen patients underwent attempted symmetric bicuspidization: 9 historical and 5 simulation-guided (Table 1). Diagnoses varied widely (Table 2), and twelve (86%) patients underwent concurrent operations at time of bicuspidization repair. Median cross-clamp time for the cohort was 74.5 (52.8,107.5) minutes. Valve configuration included unicuspid (*n* = 2), bicuspid (*n* = 6), tricuspid (*n* = 4), and quadricuspid (*n* = 2). Outlet valve type included native aortic valve (*n* = 10), neo-aortic valve after arterial switch (*n* = 2), and native truncal valve (*n* = 2), with repair indication predominantly regurgitation (*n* = 13/14, 93%). Autologous pericardial patch was used as part of valve repair in three (21%) patients, all in the historical group.

**Table 1:** Patient baseline characteristics.

Characteristic	Overall (*n* = 14)	Historical (*n* = 9)	Simulation-Guided (*n* = 5)	p
Age at surgery (years)	5.9 (2.7,11.9)	9.9 (4.8,16.2)	2.9 (2.4,6.5)	0.16
Preop mean gradient (mmHg)	5.8 (2.8,9.1)	6.5 (2.7,10.6)	5.1 (3.0,8.5)	0.55
Preop regurgitation grade	2.0 (2.0,2.5)	2.0 (1.5,2.5)	2.0 (2.0,2.5)	0.73
STJ diameter (mm)	15.5 (14,20)	15 (14,20)	16 (15,20)	1.00
Valve Configuration				0.47
Unicuspid	2 (14%)	2 (22%)	0 (0%)	
Bicuspid	6 (43%)	3 (34%)	3 (60%)	
Tricuspid	4 (29%)	2 (22%)	2 (40%)	
Quadricuspid	2 (14%)	2 (22%)	0 (0%)	
Valve Type				0.10
Native aortic valve	10 (72%)	8 (89%)	2 (40%)	
Neo-aortic valve	2 (14%)	0 (0%)	2 (40%)	
Truncal valve	2 (14%)	1 (11%)	1 (20%)	
Repair Indication				1.00
Regurgitation	13 (93%)	8 (89%)	5 (100%)	
Stenosis	0 (0%)	0 (0%)	0 (0%)	
Mixed	1 (7%)	1 (11%)	0 (0%)	
Patch Used in Repair				0.26
Yes	3 (21%)	3 (33%)	0 (0%)	
No	11 (79%)	6 (67%)	5 (100%)	
FELAD ratio				—
1.0	1 (7%)	1 (11%)	0 (0%)	
1.5	1 (7%)	0 (0%)	1 (20%)	
1.6	4 (29%)	0 (0%)	4 (80%)	
unknown	8 (57%)	8 (89%)	0 (0%)	
Total follow-up (days)	492 (91,1096)	948 (11,2389)	455 (326,529)	0.84

Values are median (IQR) or n (%). *P*-values from Wilcoxon rank-sum (continuous) or Fisher exact test (categorical). Regurgitation grading: trivial = 0.5, mild = 1, mild–mod = 1.5, moderate = 2, mod–severe = 2.5, severe = 3.

FELAD, free-edge length to aortic diameter; STJ, sinotubular junction.

**Table 2:** Patient description table.

ID	Age	STJ (mm)	FELAD	Acute Gradient (mmHg)	Latest Gradient (mmHg)	Clamp time	Diagnoses	Concurrent Procedures
*Historical*								
h1	16.9	24	1.0	29.6	29.6	127	quadricuspid aortic regurgitation with ventricular dilation	none
h2	16.2	25.2	.	16	14.5	112	unicuspid aortic valve with mixed disease, subaortic membrane	subaortic membrane resection
h3	12.4	18	.	5	3.0	70	bicuspid aortic valve regurgitation, endocarditis complicated by mitral valve perforation	mitral valve repair
h4	2.6	12	.	14	11.8	72	quadricuspid truncal valve s/p neonatal repair, with regurgitation and conduit degradation	redo sternotomy, conduit exchange
h5	4.8	15	.	7.2	7.8	52	d-TGA s/p arterial switch with tricuspid neo-aortic regurgitation, severe neo-pulmonary regurgitation with supravalvar stenosis	pulmonary valve repair, PAR
h6	9.9	12	.	-	-	115	ToF/PA/MAPCAs with dysplastic tricuspid aortic valve s/p unifocalization and commissurotomy, with conduit failure and severe aortic regurgitation	pulmonary conduit exchange
h7	1.0	14	.	15	50.0	77	neonatal bicuspid aortic stenosis s/p balloon valvotomy with regurgitation, subaortic membrane, supravalvar ridge, ascending aortic aneurysm	subaortic and supravalvar resection, reduction aortoplasty
h8	17.7	20	.	13	13.0	43	unicuspid aortic regurgitation with ventricular dilation	none
h9	5.3	14	.	7.3	3.3	17	ToF/PA s/p m-BTT takedown and intracardiac repair, with bicuspid aortic regurgitation, conduit failure, and branch pulmonary artery stenosis	PAR, conduit exchange
*Simulation-Guided*								
s1	10.6	20	1.6	9.6	9.6	94	DORV s/p arterial switch and arch repair w/ LVOTO and bicuspid neo-aortic regurgitation	subaortic membrane resection
s2	2.4	15	1.6	3.2	3.3	181	DORV mild pulmonary stenosis s/p pulmonary valvuloplasty and PAB w/ bicuspid aortic regurgitation, posterior mitral ridge, tricuspid valve regurgitation	PDA ligation, ASD repair, PAB takedown, Rastelli baffle, mitral, tricuspid, and pulmonary valve repair
s3	2.9	23.9	1.6	11.8	9.0	36	truncus arteriosus with pulmonary artery stenosis s/p neonatal repair and left PAR, with bicuspid truncal valve regurgitation and ventricular dilation	PAR with homograft patch augmentation, conduit exchange
s4	0.6	11	1.5	10.4	3.0	89	ToF/PA/MAPCAs with tricuspid aortic regurgitation	unifocalization of right and left lungs, VSD repair, conduit
s5	6.5	16	1.6	6.2	2.5	55	interrupted aortic arch with VSD s/p arch repair and VSD closure s/p Yasui with tricuspid neo-aortic regurgitation and conduit failure	conduit exchange, branch PAR

Descriptive table of all patients in the historical and simulation-guided groups.

ASD, atrial septal defect; DORV, double outlet right ventricle; d-TGA, d-looped transposition of the great arteries; FELAD, free-edge length-to-aortic diameter ratio; LVOTO, left ventricular outflow tract obstruction; MAPCAs, major aortopulmonary collateral arteries; m-BTT, modified Blalock-Taussig-Thomas shunt; PAB, pulmonary artery band; PAR, pulmonary artery reconstruction; PDA, patent ductus arteriosus; STJ, sinotubular junction; ToF/PA, Tetralogy of Fallot with pulmonary atresia.

Median sinotubular junction diameter was 15.5 (14.0, 20.0) mm. Preoperative mean transvalvular gradient and regurgitation grade were not significantly different between groups (*p* > 0.05). The FELAD was 1.6 in four patients and 1.5 in one of the simulation-guided group, while all but one patient in the historical group did not have a documented measurement of the free-edge length. The one historical patient had a FELAD ratio of 1.0 (free-edge length 24 mm, sinotubular junction 24 mm) and experienced early postoperative stenosis requiring in-hospital valve replacement with pulmonary autograft.

There were zero deaths in the overall cohort over median follow-up of 492 (91.2, 1096) days (Table 3). Five (56%) patients in the historical group underwent reoperation or replacement, while there were zero events in the simulation-guided group (Figure 2) (follow-up of 948 vs. 455 days, *p* = 0.84). Indication for reoperation or replacement was stenosis in two patients (40%), regurgitation in two (40%), and mixed stenosis and regurgitation in the one patient who required in-hospital valve replacement mentioned above (20%). Three out of the five patients (60%) who underwent reoperation or replacement had autologous pericardial patch used as part of the valve repair.

**Table 3:** Outcomes across historical and simulation-guided groups.

Outcome	Historical (*n* = 9)	Simulation-Guided (*n* = 5)	p
Reoperation, *n* (%)	5 (56%)	0 (0%)	0.09
Death, *n* (%)	0 (0%)	0 (0%)	1.00
Repeat bypass, *n* (%)	3 (33%)	0 (0%)	0.26
Bypass runs, n	1.3 ± 0.5	1.0 ± 0	0.16
Cross-clamp time (min)	76.1 ± 36.4	91.0 ± 55.8	0.74
Acute mean gradient (mmHg)	13.4 ± 7.7	8.2 ± 3.5	0.14
Acute regurgitation grade	1.4 ± 0.4	1.3 ± 0.4	0.75
Latest mean gradient (mmHg)	16.6 ± 15.9	5.5 ± 3.5	0.08
Latest regurgitation grade	1.7 ± 0.8	1.1 ± 0.5	0.15
Length of stay (days)	7.2 ± 6.1	10.6 ± 9.4	0.31
Total follow-up (days)	1,235.1 ± 1,470.9	514.6 ± 331.3	0.84

Values are mean ± SD or *n* (%). *P*-values from Wilcoxon rank-sum (continuous) or Fisher exact test (categorical). Regurgitation grading: trivial = 0.5, mild = 1, mild–mod = 1.5, moderate = 2, mod–severe = 2.5, severe = 3.

**Figure 2:**
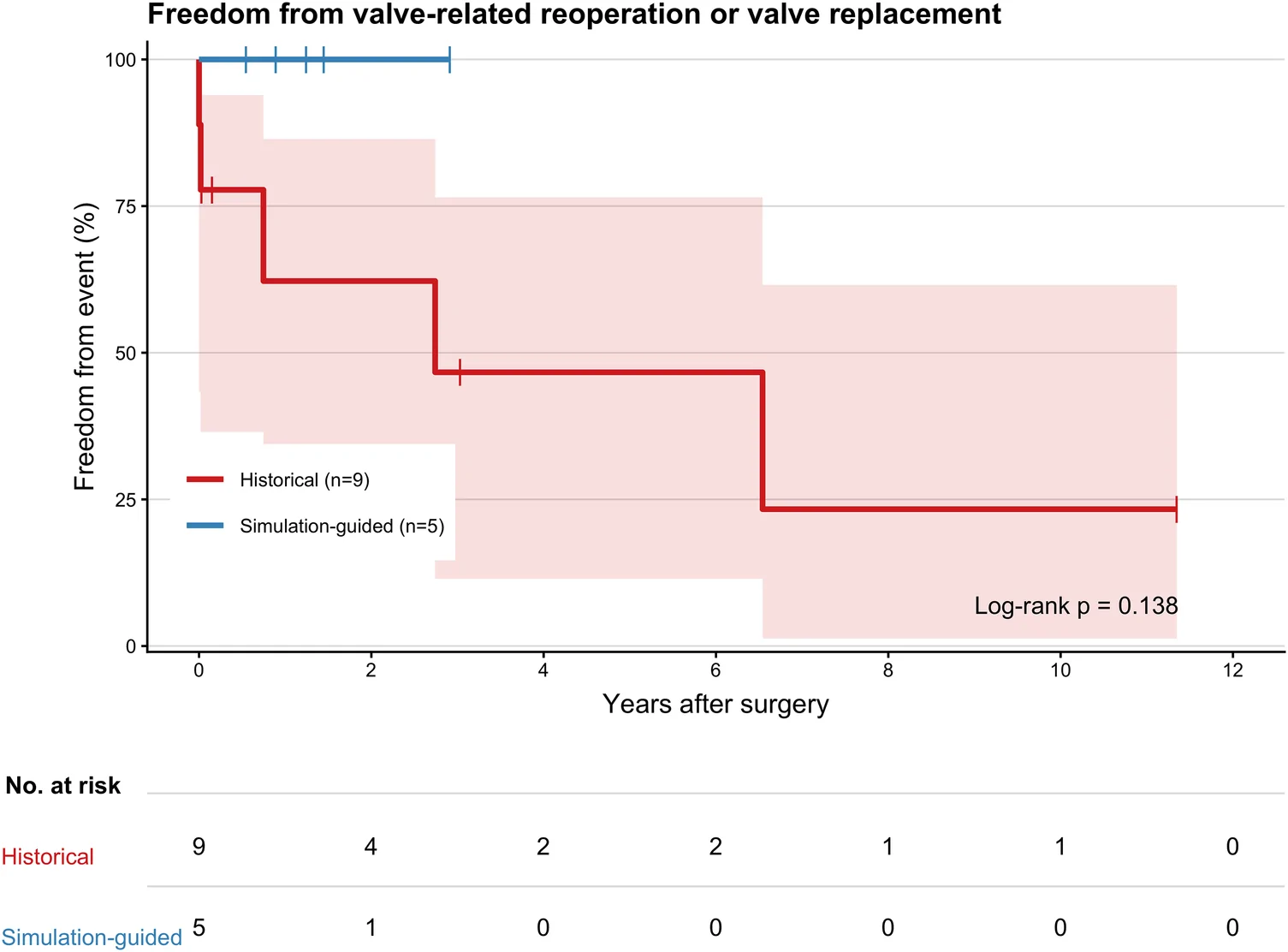
Kaplan–meier curve for freedom from valve-related reoperation or valve replacement after surgery, comparing historical repairs with simulation-guided repairs. Patients without an event were censored at the date of last follow-up. Shaded bands represent 95% confidence intervals. Tick marks indicate censored observations. The number of patients at risk is shown below the *x*-axis. Groups were compared using the log-rank test. Numbers at risk are presented at 0, 2, 4, 6, 8, 10, and 12 years and are vertically aligned with the corresponding *x*-axis time points.

Three (33%) patients in the historical group required repeat bypass run at time of index valve repair, compared to zero patients in the simulation-guided group (*p* = 0.26). Cross-clamp time was not significantly different between groups (historical 76.1 ± 36.4, simulation-guided 91.0 ± 55.8, *p* = 0.74). Length of stay was also not significantly different (historical 7.2 ± 6.1, simulation-guided 10.6 ± 9.4, *p* = 0.32).

In the acute period, postoperative mean gradients were 13.4 ± 7.7 mm Hg for the historical and 8.2 ± 3.5 mm Hg for the simulation-guided group (*p* = 0.14) (Figure 3). Mean acute regurgitation grade was 1.4 ± 0.4 for the historical and 1.3 ± 0.4 for the simulation-guided (*p* = 0.77). For the latest echocardiogram prior to last follow-up or replacement, gradients were 16.6 ± 15.9 mm Hg for historical and 5.5 ± 3.5 mm Hg for the simulation-guided group (*p* = 0.08). Mean latest regurgitation grade was 1.7 ± 0.8 for the historical and 1.1 ± 0.5 for the simulation-guided (*p* = 0.19). Median time to latest echocardiogram was 511.5 (18.5, 1,355.25) days for historical and 326.0 (319, 504) for simulation-guided (*p* = 0.94). In sensitivity analysis excluding the three historical patch-augmentation patients, valve-related reoperation or replacement occurred in 2 of 6 historical patients and 0 of 5 simulation-guided patients (Fisher exact *p* = 0.46); the log-rank comparison remained nonsignificant (*p* = 0.36). Acute and latest mean-gradient comparisons were likewise nonsignificant (*p* = 0.60 and *p* = 0.34, respectively).

**Figure 3:**
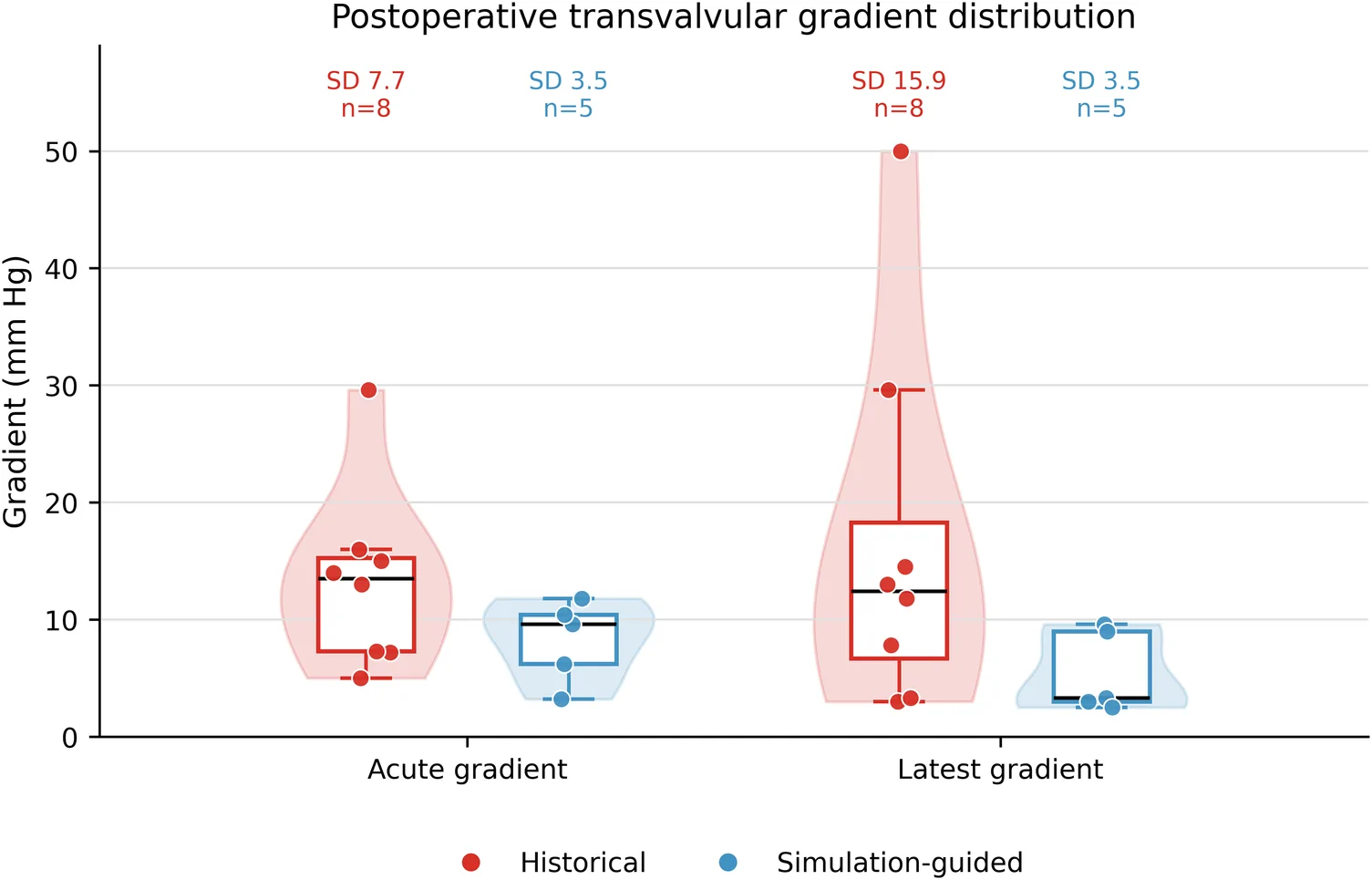
Distribution of postoperative transvalvular gradients in the historical and simulation-guided groups. Violin plots show the distribution of acute and latest gradients, with embedded boxplots showing the median and interquartile range. Individual points represent patients. Text annotations indicate group-specific standard deviation and sample size for each time point.

## Comment

Computational simulation is becoming an increasingly important tool in the armamentarium of the modern congenital heart surgeon—so too, the partnership with engineers who design these powerful tools. The low prevalence and high variability of congenital lesions offer a uniquely rich environment for customized treatment planning tools, and methods have been tailored to generate realistic heart model variations to deepen testing (20). Nevertheless, simulations remain imperfect, and clinical implementation of valve-based models—which are notoriously more challenging due to their complex, intricate structure—are particularly rare. Despite utilizing a generalized valve-based model, the current study suggests that simulation-guided design tools may benefit outcomes in clinical scenarios still dependent on non-standardized, subjective decision-making.

Freedom from valve-related reoperation was not significantly different despite a divergence in curves—with 50% of the historical cohort requiring reoperation versus none in the simulation-guided group at 1,000 days after surgery. Certainly, short follow-up of the simulation-guided group and generally small cohort numbers underpowered comparison. But in the immediate perioperative period, where follow-up is more comparable, there was no need for multiple valve-related bypass runs in the simulation-guided group, while there was a need in one-third (*n* = 3/9) of the historical group, with one of these resulting in valve replacement rather than repeat repair attempt. Despite this, cross-clamp time was similar between groups. This is likely secondary to the heterogeneity in concurrent diagnoses and operations between groups, as avoiding the need for an additional bypass run, by definition, decreases overall cross-clamp time for a given patient. Consistent with prior studies (21, 22), use of patch material (autologous pericardium in all cases) was prevalent in cases requiring reoperation or replacement (60%, *n* = 3).

Patch augmentation was confined to the historical group and was present in three of the five patients who experienced reoperation or replacement. Confounding by repair technique cannot be excluded; however, excluding the three patch-augmentation patients did not materially change the direction or statistical significance of the primary-outcome or gradient comparisons in sensitivity analysis. These estimates remain imprecise given the small cohort.

While FELAD was 1.5–1.6 in all simulation-guided patients, the FELAD ratio was missing for all but one patient in the historical cohort. Interestingly, the bicuspidized valve in that patient was sized to a ratio of 1.0 and subsequently required in-hospital replacement due to significant acute postoperative stenosis, as expected per prior work (3–5) While it is possible that several of the historical patients were sized around 1.57, it is unlikely the ratio would be as preserved in the historical group given the subjective approach applied. Importantly, all valves in the simulation-guided group were sized only in reference to the free-edge length as opposed to the geometric and effective heights, meaning all observed results are attributable to a prespecified, standardized FELAD-based approach.

While statistically significant differences in gradients were not observed, standard deviation in the historical group was over twice as high as the simulation-guided group in the acute setting (13.4 ± 7.7 vs. 8.2 ± 3.5) and over four times as high in the latest echocardiograms (16.6 ± 15.9 vs. 5.5 ± 3.5). Importantly, total follow-up time was not significantly different between groups, and the measured time to latest echocardiogram was even closer (median 512 vs. 326 days, *p* = 0.94). Furthermore, the variation in acute gradient (within first two postoperative months) was captured well within the follow-up range for both groups. Given the simulation-guided group adopted a non-subjective, standardized approach, the smaller variation in gradient may point to improved consistency of repair.

As with all before-after studies there is an inherent limitation in potential confounding from time-based variables. Importantly, the same surgeons were present in both time periods, and there was no institutional change in approach to symmetric bicuspidization, or its indications for use or for reoperation. The lack of difference in pre-operative gradients and regurgitation grade, as well as the non-overlap of post-operative gradients in the simulation-guided group with those of patients who underwent reoperation for stenosis in the historical group, support this claim. The slight temporal overlap of the two groups and inclusion of all symmetric bicuspidization attempts in the study period also tempers the risk of confounding, though only partially.

Another limitation is that the previous simulation study focused on cases in which the annular and sinotubular junction diameters were equal, which may vary in clinical practice. Since the sinotubular junction is straightforward to modify surgically, the combined interplay of these diameters and free edge length should be studied in future work. Differences between the STJ and basal-ring diameters may therefore alter the effective FELAD relationship and limit direct generalization of the simulated target. Moreover, although the present cohort comprised pediatric and young patients with congenital outlet-valve disease, the measurement-based FELAD principle may also be applied to adult patients, though bicuspidization repair is not commonly used in adults and its applicability may depend on leaflet mobility, tissue quality, calcification, and root geometry. Dedicated clinical validation in adults is warranted.

While exploratory in scope, the current study presents a rare example of successful translation of simulation-guided principles in complex valve repair with promising early results. Longer follow-up and larger cohorts will be necessary, and further refinements explored, particularly in adoption of patient-specific modeling. Computational models in standardizing complex valve repair appear feasible and will prove increasingly important moving forward.

## Data Availability

The raw data supporting the conclusions of this article are available on request to the corresponding author.
